# Short stature, platyspondyly, hip dysplasia, and retinal detachment: an atypical type II collagenopathy caused by a novel mutation in the C-propeptide region of *COL2A1*: a case report

**DOI:** 10.1186/s12881-016-0357-4

**Published:** 2016-12-12

**Authors:** Apiruk Sangsin, Chalurmpon Srichomthong, Monnat Pongpanich, Kanya Suphapeetiporn, Vorasuk Shotelersuk

**Affiliations:** 1 Center of Excellence for Medical Genetics, Department of Pediatrics, Faculty of Medicine, Chulalongkorn University, Bangkok, 10330 Thailand; 2 Excellence Center for Medical Genetics, King Chulalongkorn Memorial Hospital, the Thai Red Cross Society, 10330 Bangkok, Thailand; 3 Department of Orthopedics, Faculty of Medicine, Chiang Mai University, 50200 Chiang Mai, Thailand; 4 Interdepartment Program of Biomedical Sciences, Faculty of Graduate School, Chulalongkorn University, 10330 Bangkok, Thailand; 5 Department of Mathematics and Computer Science, Faculty of Science, Chulalongkorn University, 10330 Bangkok, Thailand; 6 Program in Bioinformatics and Computational Biology, Graduate School, Chulalongkorn University, 10330 Bangkok, Thailand; 7 Division of Medical Genetics and Metabolism, Department of Pediatrics, Faculty of Medicine, Chulalongkorn University, Sor Kor Building 11th floor, 10330 Bangkok, Thailand

**Keywords:** *COL2A1*, C-propeptide region, Exome sequencing, Type II collagenopathies

## Abstract

**Background:**

Heterozygous mutations in *COL2A1* create a spectrum of clinical entities called type II collagenopathies that range from *in utero* lethal to relatively mild conditions which become apparent only during adulthood. We aimed to characterize the clinical, radiological, and molecular features of a family with an atypical type II collagenopathy.

**Case presentation:**

A family with three affected males in three generations was described. Prominent clinical findings included short stature with platyspondyly, flat midface and Pierre Robin sequence, severe dysplasia of the proximal femora, and severe retinopathy that could lead to blindness. By whole exome sequencing, a novel heterozygous deletion, c.4161_4165del, in *COL2A1* was identified. The phenotype is atypical for those described for mutations in the C-propeptide region of *COL2A1*.

**Conclusions:**

We have described an atypical type II collagenopathy caused by a novel out-of-frame deletion in the C-propeptide region of *COL2A1*. Of all the reported truncating mutations in the C-propeptide region that result in short-stature type II collagenopathies, this mutation is the farthest from the C-terminal of *COL2A1*.

## Background

Patients with *COL2A1* mutations are collectively called type II collagenopathies. Missense mutations or in-frame derangement in the triple-helical region cause a phenotype on the spondyloepiphyseal dysplasia (SED) spectrum from lethal SED including achondrogenesis type II (ACG2; OMIM# 200610) and hypochodrogenesis through spondyloepiphyseal dysplasia congenita (SEDC; OMIM#183900), while mutations in the triple helical or N-propeptide regions cause Stickler syndrome type I (STL1; OMIM# 108300) or Kniest dysplasia (OMIM# 156550) [1]. Unlike mutations in the triple-helical or N-propeptide regions, those in the C-propeptide region generally produce atypical phenotypes such as platyspondylic lethal skeletal dysplasia, Torrance type (PLSDT# OMIM 151210) [2], spondyloperipheral dysplasia (SPPD) (OMIM# 271700) [3], vitreoretinopathy with phalangeal epiphyseal dysplasia (VPED) [4], avascular necrosis of the femoral head (ANFH; OMIM# 608805) [5], or early-onset osteoarthritis (OA) [6]. Here, we describe a family with atypical features of type II collagenopathies caused by a novel mutation in *COL2A1*. As far as we know, this truncating mutation in the C-propeptide region is the farthest one from the 3’ end of the gene that causes a disease with short stature, suggesting the existence of the mutant protein.

## Case presentation

### Subjects

We studied a Thai family with skeletal dysplasia who attended the Genetics Clinic at the King Chulalongkorn Memorial Hospital, Bangkok, Thailand. The medical data, pedigree, physical examinations, and laboratory results were recorded. The written informed consent and parental consent (for the proband) was obtained after explanation of the possible consequences of this study.

### Genomic DNA preparation and whole-exome sequencing

To perform genetic analysis, genomic DNA was isolated from peripheral blood leukocytes using a Puregene Blood kit (Qiagen, Hilden, Germany). The genomic DNA was sent to Macrogen, Inc. (Seoul, South Korea) for whole-exome sequencing (WES). DNA was captured using a SureSelect Human All Exon version 4 kit (Agilent Technologies, Santa Clara, CA) and sequenced on a Hiseq2000 instrument. Base calling was performed and quality scores were analyzed using Real Time Analysis software version 1.7. Sequence reads were aligned against the University of California Santa Cruz human genome assembly hg19 using Burrows-Wheeler Alignment software (bio-bwa.sourceforge.net/). Single-nucleotide variants (SNVs) and insertions/deletions (Indels) were detected by SAMTOOLS (samtools.sourceforge.net/) and annotated against dbSNP & the 1000 Genomes Project. After quality filtering, we looked for variants located in the coding regions of known skeletal dysplasia genes for all potential pathogenic SNVs and Indels. Variant calling exclusion criteria were (a) coverage <10×; (b) quality score <20; (c) minor allele frequency ≥1% in the 1000 Genomes Project; and (d) non-coding variants and synonymous exonic variants. The remaining variants were subsequently filtered out if they were present in our in-house database of 165 unrelated Thai exomes. The variants were confirmed by PCR and Sanger sequencing.

Existing SNVs or known pathogenic mutations were filtered out using the Human Gene Mutation Database (http://www.hgmd.cf.ac.uk/ac/index.php) and the Exome Aggregation Consortium database (exac.broadinstitute.org).

## Results

A 20 month-old male (IV:3; Fig. 1) is the first child of a non-consanguineous couple. His mother had miscarriages in the first trimester of the two previous pregnancies. The causes of both miscarriages were unknown. He was born at term by normal delivery with a birth weight of 2,850 g (10^th^ centile) and a length of 45 cm (<3^rd^centile, −4 SD). Physical examination revealed short stature, flattened face, cleft palate, micrognathia, short neck, and umbilical hernia (Fig. 2a). A radiograph obtained at age 20 months showed oval-shaped vertebral bodies (Fig. 2b). Ossification of the femoral head was absent. Long bones showed short broad tubular shape with metaphyseal flaring (Fig. 2c). Other ossification centers appeared age-appropriate (Fig. 2c and d). Hands and feet radiographs were normal (Fig. 2e and f). His eyes examination and audiometry showed no abnormality. He was given a clinical diagnosis of SEDC.

**Fig. 1:**
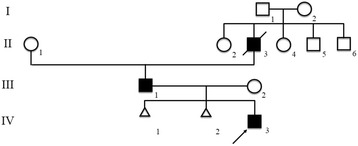
Pedigree of the index family. The arrow indicates the proband

**Fig. 2:**
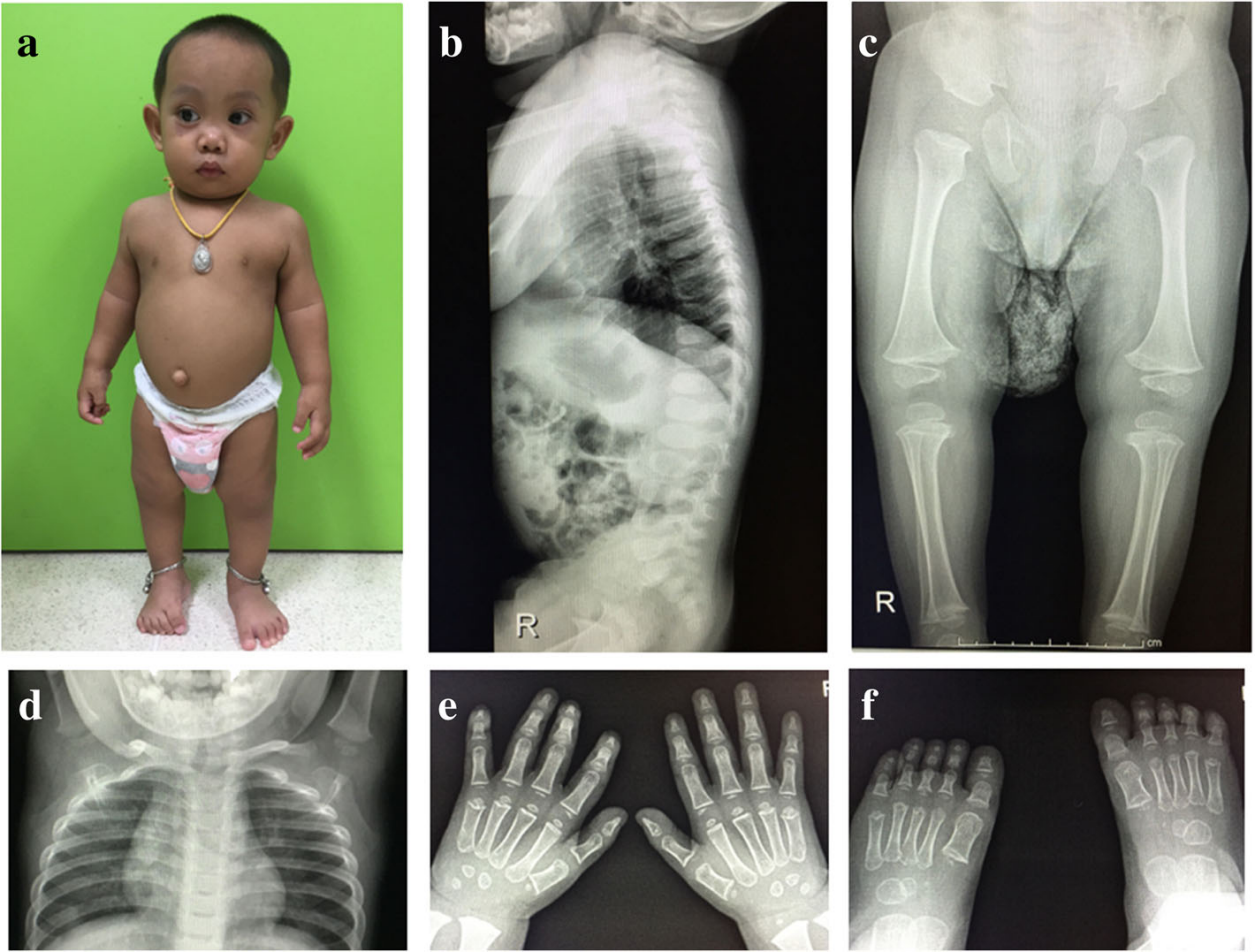
**a** Photograph of the proband (IV: 3) showing flattened facial profile, micrognathia, short neck, and short trunk. **b** Whole spine lateral radiograph showing oval-shaped vertebral bodies. **c** Pelvis, femur, and tibial AP radiograph showing retarded ossification of the femoral heads while other ossification centers are age-appropriate. **d** Chest radiograph showing age-appropriate ossification centers at humeral heads and greater tuberosities of humeri. **e**, **f** Hand and foot AP radiographs are normal

**Fig. 3:**
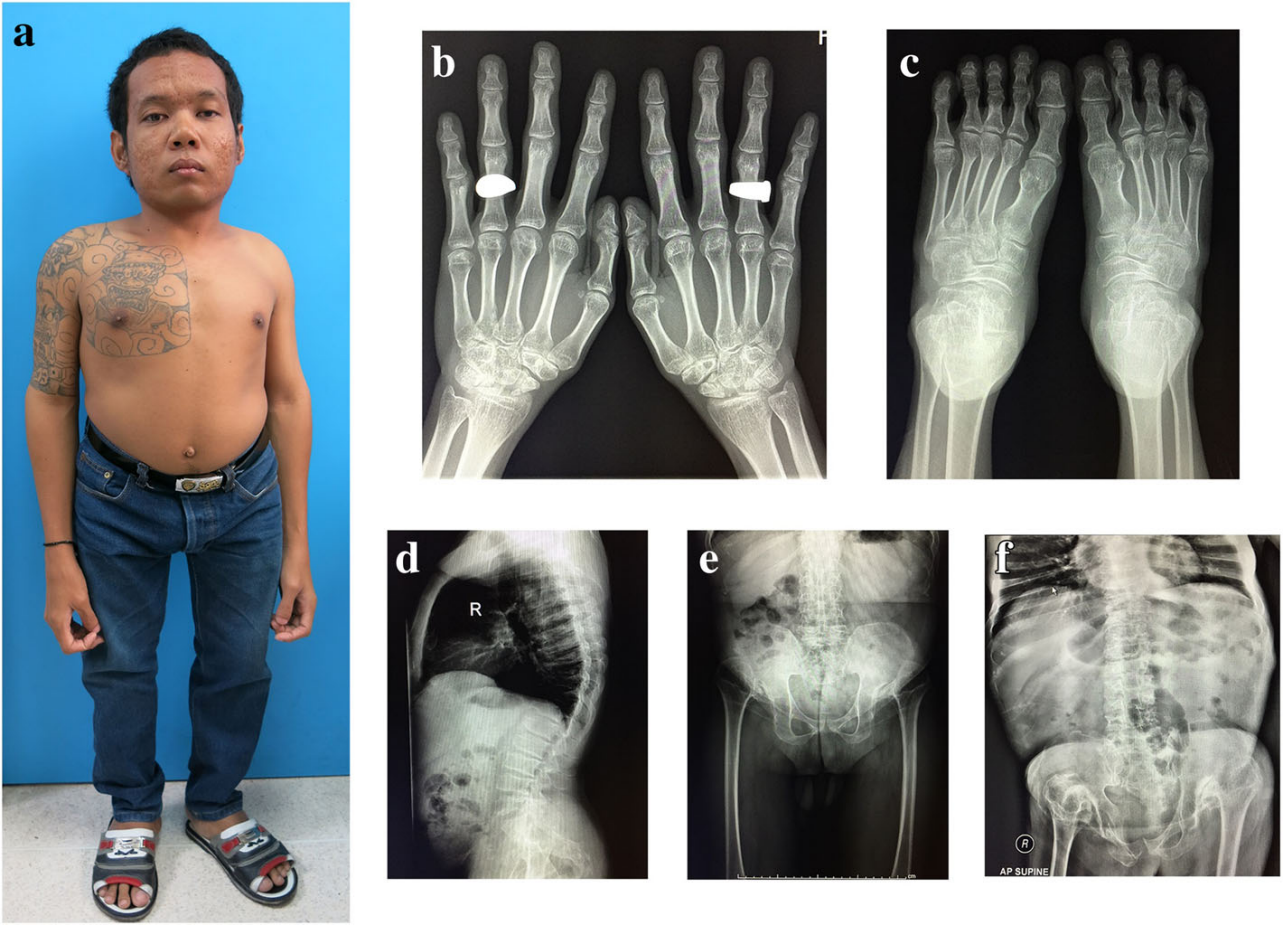
**a** Photograph of proband’s father (III: 1) showing disproportionate short stature and barrel-shaped chest. **b**, **c** Hand and feet radiographs are normal without brachydactyly. **d** Lateral radiograph of the thoracolumbar spine shows platyspondyly with kyphotic deformity. **e** Pelvis AP radiograph showing severe hip dysplasia and dislocation. **f** Thoracolumbar spine and pelvis AP radiographs of the proband’s grandfather also show platyspondyly and severe hip dysplasia and dislocation

The proband’s father (III:1; Fig. 1) is a 26 year-old man. He had short trunk dwarfism with a height of 125 cm (−9 SD). He had a barrel-shaped chest, hyperlordosis of the lumbar spine, and flexion contracture of both hips (Fig. 3a). Hands and feet, including radiographs, were apparently normal (Fig. 3b and c). A radiograph of the thoracolumbar spine showed flattened vertebral bodies with kyphotic deformity (Fig. 3d). Severe dysplasia of the bilateral proximal femoral epiphyses and hip dislocation were observed (Fig. 3e). Generalized osteopenia was noted. He was blind in the left eye since he was 8 years old, while his right eye had severe myopia and retinal detachment. He also had umbilical hernia when he was young but it spontaneously resolved.

The proband’s grandfather (II:3; Fig. 1) had short stature (−8 SD). He also had a barrel-shaped chest, hyperlordosis of the lumbar spine and flexion contracture of both hips. A radiograph of the thoracolumbar spine and pelvis showed flattened vertebral bodies, severe dysplasia of the bilateral proximal femoral epiphysis and hip dislocation (Fig. 3f). His hands and feet were normal. His eyes were reported normal but had never been formally evaluated. He died at the age of 54 because of septicemia after a nephrostomy as treatment for obstructive uropathy from ureteric stones. A blood sample was not available for mutation analysis.

**Fig. 4:**
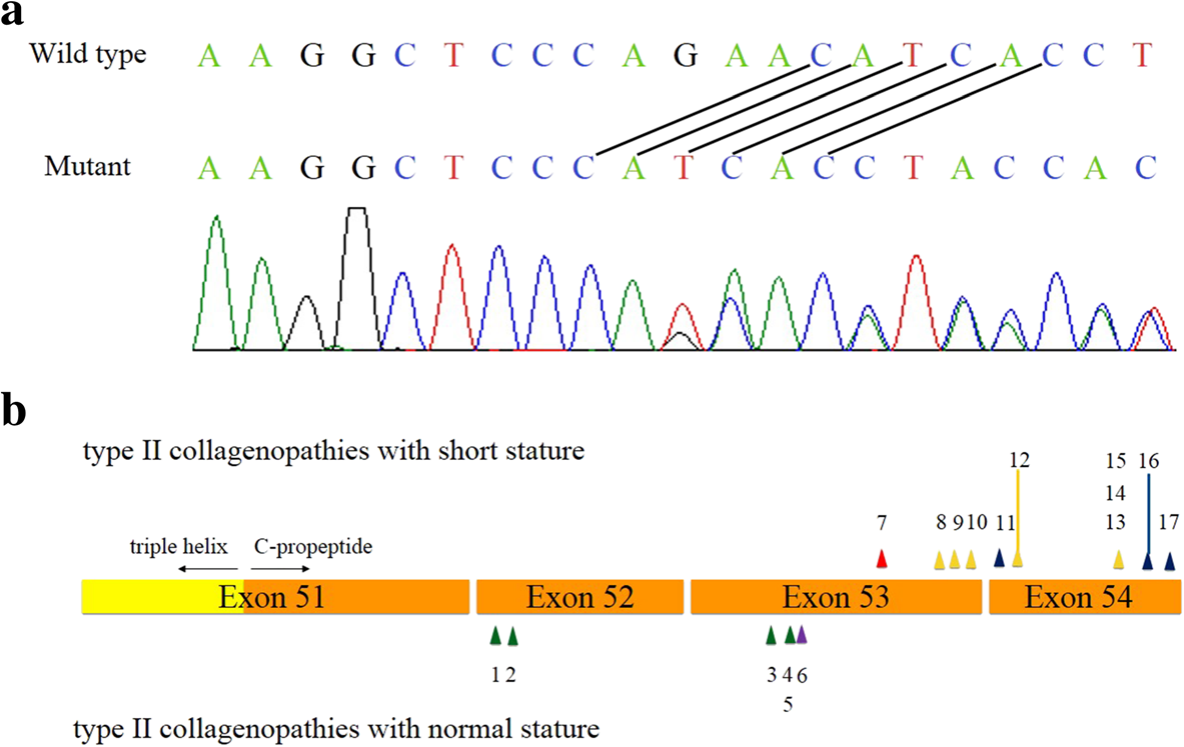
Mutation analysis. **a** Direct sequencing shows that the proband is heterozygous for a five nucleotide out-of-frame deletion (NM_001844.4: c.4161_4165del:p.Gln1387His*fs30). **b** Map of the mutations in the C-propeptide region of *COL2A1.* Truncating mutations shown above the gene lead to collagenopathies with short stature, while those shown below the gene lead to disorders with normal stature. The arrowheads points to the last amino acid residue of the predicted truncated COL2A1. *Green*: STLI; *purple*: early-onset OA; *red*: our mutation; *yellow*: SPPD; *blue*: PLSDT. Numbers correspond to Table 2

Whole-exome sequencing (WES) of the proband revealed a five-nucleotide out-of-frame deletion (NM_001844.4: c.4161_4165del:p.Gln1387Hisfs*30) in *COL2A1*. PCR and Sanger sequencing using leukocyte-derived DNA from the proband (IV:3) and his father (III:1) confirmed that both were heterozygous for the mutation (Fig. 4a). This mutation has not been reported previously in the Human Genome Mutation Database or the Exome Aggregation Consortium database. In addition, it was not present in our in-house exome database of 165 Thai individuals.

## Conclusions

Major clinical and radiographic features in our patients include short stature with platyspondyly, flat midface, Pierre Robin sequence, hip dysplasia, and retinal detachment. The major extracellular structural protein of these affected organs is collagen type II. This led us to hypothesize that the etiologic mutation was in *COL2A1*. WES identified a five-base pair deletion in this gene. PCR and Sanger sequencing confirmed that the proband (IV:3) and his father (III:1) harbored a heterozygous mutation, c.4161_4165del, in *COL2A1.* This out-of-frame deletion is in the C-propeptide region and is expected to lead to a protein truncation, p.Gln1387Hisfs*30.

Although our patients have a mutation in C-propeptide region of *COL2A1*, the phenotype is atypical for those described for the mutations in C-propeptide region. Previous reported mutations in the C-propeptide region of collagen type II lead to one of the six entities: PLSDT, SPPD, VPED, ANFH, STL1, or early-onset OA. However, the clinical features of this family are different from all these six diseases (Table 1).

**Table 1:** Clinical features of type II collagenopathies resulting from mutations in the C-propeptide region of *COL2A1*, compared with this family

	PLSDT	SPPD	VPED	ANFH	STLI	Early-onset OA	Proband (IV:3)	Father (III:1)	Grandfather (II:3)
Lethal	+	-	-	-	-	-	-	-	-
Short stature	+	+	-	-	-	N/A	+	+	+
Delayed ossification	+	+	-	N/A	-	N/A	+ (hip only)	N/A	N/A
Platyspondyly	+	+	-	-	+	N/A	+	+	+
Metaphyseal involvement	+	+	-	-	-	N/A	+	+	N/A
Epiphyseal involvement	+	+	+	+ (hip only)	+	N/A	+	+	+
Early onset arthritis	N/A	+	+	+ (hip only)	+	+	N/A	-	N/A
Brachydactyly	+	+	+	-	-	N/A	-	-	-
Osteopenia/osteoporosis	N/A	N/A	N/A	+	+	N/A	N/A	+	+
Early onset myopia	-	+	-	-	+	N/A	-	+	N/A
Retinal detachment/tear	-	+	+	-	+	N/A	-	+	N/A
Glaucoma	-	-	-	-	+	N/A	-	+	N/A
Vitreal abnormalities	+	+	+	-	+	N/A	-	-	N/A
Hearing loss	-	+	-	-	+	N/A	-	-	-
Flattened facial profile	+	+	-	-	+	N/A	+	-	-
Pierre-Robin sequence	+	N/A	-	-	+	N/A	+	-	-
Cleft lip/cleft palate/bifid uvula	-	+	-	-	+	N/A	+	-	-

*N/A* not available, *PLSDT* platyspondylic lethal skeletal dysplasia, Torrance type, *SPPD* spondyloperipheral dysplasia, *VPED* vitreoretinopathy with phalangeal epiphyseal dysplasia, *ANFH* avascular necrosis of the femoral head, *STL1* Stickler syndrome type I, *OA* osteoarthritis

PLSDT was excluded by absence of wafer-thin platyspondyly, small round scapulae, and brachydactyly in our patients. Moreover, PLSDT patients generally die at birth [7] but our patients live into adulthood. An important diagnostic feature of SPPD, brachydactyly [3], excluded the diagnosis of SPPD. Short stature with platyspondyly without phalangeal epiphyseal dysplasia excluded VPED from the diagnosis [4]. ANFH patients have flattened femoral heads with signs of premature osteoarthritis. However, the femoral heads of proband’s father (III:1) and grandfather (II: 3) were totally absent causing bilateral hip dysplasia and dislocation. Another important difference is the fact that ANFH patients do not have short stature, platyspondyly or retinal abnormalities [5], while our patients do. Patients with STLI usually have normal stature and premature OA of many joints [8, 9], while all our patients (III:1 and II:3) had short stature (−8 to −9 SD at adulthood). Early-onset OA patients should present with premature OA of joints without other skeletal abnormalities, while our patients had short stature and severe dysplasia of the hips.

STL1 is caused by haploinsufficiency of *COL2A1*. All point mutations in *COL2A1* leading to STL1 are therefore expected to be subjected to nonsense-mediated mRNA decay (NMD). Heterozygous mutations leading to type II collagenopathies besides STL1 are predicted to have a dominant negative effect. Previous reports showed that truncating mutations in the C-propeptide region led either to STL1 or SPPD, when the mutant RNA did, or did not, undergo NMD, respectively (Table 2).

**Table 2:** Truncating mutations in the C-propeptide region of *COL2A1*

No.	Variant location	Nucleotide change	Predicted protein change	Clinical diagnosis	Reference
1	Exon 52	c.3906del	p.Asn1303Thrfs*9	STL1	Hoornaert et al. [12]
2	Exon 52	c.3891_3898dup	p.Ile1300Thrsfs*15	STL1	Hoornaert et al. [12]
3	Intron 52	c.4074 + 1G > T	p.Trp1348Cysfs*17	STLI	Hoornaert et al. [12]
4	Exon 52	c.3978delC	p.Asn1327Ilefs*49	STLI	Ahmad et al. [13]
5	Exon 52	c.3957del	p.Gly1320Alafs*56	STL1	Annunen et al. [14]
6	Exon 53	c.4088del	p.Asp1363Valfs*13	Early-onset OA	Barat-Houari et al. [15]
7	Exon 53	c.4161_4165del	p.Gln1387Hisfs*30	This report	
8	Exon 53	c.4300del	p.Leu1434*	SPPD	Barat-Houari et al. [15]
9	Exon 53	c.4287_4291dup	p.Tyr1431Serfs*6	SPPD	Zabel et al. [16]
10	Exon 53	c.4314C > A	p.Cys1438*	SPPD	Zankl et al. [3]
11	Exon 54	c.4335G > A	p.Trp1445*	PLSDT	Zankl et al. [2]
12	Exon 54	c.4339A > T	p.Lys1447*	SPPD	Bedeschi et al. [17]
13	Exon 54	c.4332del	p.Lys1444Asnfs*27	SPPD	Zhang et al. [18]
14	Exon 54	c.4337del	p.Gly1446Alafs*25	SPPD	Zankl et al. [3]
15	Exon 54	c.4357del	p.Arg1453Glyfs*18	SPPD	Meredith et al. [19]
16	Exon 54	c.4423C > T	p.Gln1475*	PLSDT	Zankl et al. [2]
17	Exon 54	c.4413_4416del	p.Gly1472Profs*9	PLSDT	Nishimura et al. [20]

Interestingly, our frameshift mutation, expected to create a stop codon 69 nucleotides upstream from the exon 53–54 junction, does not lead to STL1. We therefore hypothesize that the mutant RNA transcribed from the mutant *COL2A1* allele found in our patients may not undergo NMD; instead that it may produce a mutant COL2A1 protein that has a dominant negative effect, interfering with the wild-type COL2A1 and leading to the unique phenotype. In general, mRNAs harboring a premature terminal codon (PCT) 50–55 nucleotides upstream of the last exon-exon junction are efficiency degraded [10]. The NMD is signaled by the presence of the exon junction complex (EJC). However, EJCs are not equally assembled at every exon junction. Therefore, it is possible that the variation of the EJC may affect NMD efficiency and lead to efficient degradation of *COL2A1* mRNAs harboring a PTC at least 70 nucleotides upstream of the final exon-exon junction. On the contrary, mutations causing premature stop codons from 69 nucleotides upstream of the exon 53–54 junction down to the 3’ end *COL2A1* would probably not undergo NMD, have a dominant negative effect, and therefore cause more severe phenotypes of the spectrum such as SPPD and PLSDT [2, 3]. Unfortunately, tissues from our patients that would express *COL2A1* are not available to test this hypothesis. Of all the reported mutations in the C-propeptide region that result in short-stature type II collagenopathies, our mutation is the farthest truncating mutation from the C-terminal of COL2A1 (Fig. 4b, Table 2).

Our probands and his father had some different phenotypes such as facial profile, eye involvement, and cleft palate. Intra-and extrafamilial variability of type II collagenopathies has previously been observed in patients with the same mutation. For instance, in a family with VPED, a 22-year-old patient had severe premature osteoarthritis requiring hip replacement while two older siblings had only mild osteoarthritis [4]. In another family, while the mosaic mother had SPPD, her fetus suffered from the lethal PLSDT [11]. These data suggest that genetic, epigenetic, and environmental modifiers affect the clinical presentation in type II collagenopathies. Moreover, these modifiers may be other possible explanations for the atypical phenotype of the patients.

In conclusion, we report an atypical phenotype resulting from a novel truncating mutation in the C-propeptide region of *COL2A1* with prominent features including short stature, platyspondyly, hip dysplasia, and retinal detachment.

## Abbreviations

ACG2: Achondrogenesis type II
ANFH: Avascular necrosis of the femoral head
NMD: Nonsense-mediated mRNA decay.
OA: Osteoarthritis
PLSDT: Platyspondylic lethal skeletal dysplasia
SED: Spondyloepiphyseal dysplasia
SEDC: Spondyloepiphyseal epiphyseal dysplasia congenital
SPPD: Spondyloperipheral dysplasia
STL1: Stickler syndrome type I
VPED: Vitreoretinopathy with phalangeal epiphyseal dysplasia
WES: Whole-exome sequencing
